# Multi-Scale Comparison of Physicochemical Properties, Refined Structures, and Gel Characteristics of a Novel Native Wild Pea Starch with Commercial Pea and Mung Bean Starch

**DOI:** 10.3390/foods12132513

**Published:** 2023-06-28

**Authors:** Xiaojun Zhang, Ning Tang, Xin Jia, Donghui Geng, Yongqiang Cheng

**Affiliations:** Beijing Key Laboratory of Functional Food from Plant Resources, College of Food Science and Nutritional Engineering, China Agricultural University, Beijing 100083, China; zhangxj@cau.edu.cn (X.Z.); ningtang@cau.edu.cn (N.T.); xinjia@cau.edu.cn (X.J.); gengdonghui08@163.com (D.G.)

**Keywords:** common vetch starch, structure, gel, in vitro digestion

## Abstract

In the present study, the morphology, refined structure, thermal properties, and dynamic rheological, texture, and digestive properties of common vetch starch, a potential new type of legume starch, were systematically investigated, and compared with commercially available pea and mung bean starch. The results showed that the composition and chemical structure of common vetch starch were similar to the pea and mung bean starch. However, the amylose content (35.69), A-chain proportion (37.62), and relative crystallinity (34.16) of common vetch starch were higher, and the particle size and molecular weight (44,042 kDa) were larger. The value of pasting properties and enthalpy change (ΔH) of gelatinization of common vetch starch was lower and higher than mung bean and pea starch, respectively, and a lower swelling power and pasting index indicate that common vetch starch had higher hot-paste and cold-paste stability. In addition, common vetch starch gel exhibited good rheology, cohesiveness, and anti-digestive properties. These results provide new insights into the broader application of common vetch starch.

## 1. Introduction

The legume is one of the most important economic crops throughout the world [1] and has received increasing attention due to its nitrogen fixation and potential for promotion of sustainable development [2,3], especially the wild pea, such as Bitter vetch (*Vicia sativa* L.) [4,5], the Faba bean, and the Horse bean (*Vicia faba* L.) [6]. And wild pea can not only provide rich nitrogen resources as a field but its seeds are rich in starch and can be used as a food source of carbohydrates [5,6,7]. Recent research has explored the seeds of the wild pea (e.g., common vetch and the faba bean) as a source of starch, protein, and active substances [8,9].

Common vetch (*Vicia sativa* L.), a wild pea cultivated in Turkey, Australia, and China [10], can grow in extreme environments and increase the yield of other crops cultivated as intercrops [11]. With the development of breeding, the yield of common vetch is increasing and *Longjian* was selected for cultivation in China, which has the potential to become a commercial seed resource. As an important resource of starch and protein, the resources of the pea are relatively poor compared to cereal and tuber, especially the resource of commercial starches. However, most research has focused on the use of common vetch as forage [12], and few studies have been concerned about its starch extraction and characterization.

Studies have shown that the carbohydrate content of common vetch seeds is more than 50% [13]. The amylose content and thermal and pasting properties of common vetch starch were different compared with pea starch [8]. However, the refined structure and gel properties of common vetch starch have not been elucidated yet, which are the limiting factors for further commercial application of starch [14,15]. As far as we know, there are no studies on the common vetch starch of the *Longjian* variety.

Therefore, the physicochemical compositions, morphology, and refined structure of common vetch (*Longjian*) starch were investigated and compared with industrialized pea starch and mung bean starch. And the thermal and pasting properties, gel viscosity and texture properties, and digestibility of the common vetch starch, which are most concerned with starch processing, were further studied. These results not only give a better understanding of common vetch starch but provide detailed parameters for its industrial application.

## 2. Materials and Methods

### 2.1. Materials

Mung bean starch and pea starch were purchased from Shandong Yantai Shuangta Company (Yantai, Shandong Province, China). Common vetch starch was extracted by lactic acid bacteria fermentation, referring to our previous method [16]. Common vetch seeds were washed before starch extraction, and then mixed with distilled water in a ratio of 1:4 and soaked for 4 h. The common vetch seeds slurry was prepared by grinding the soaked seeds with a homogenizer and collecting through a 0.1 mm sieve. Then, the starch slurry was mixed with cultured lactic acid bacteria isolated from the skin of the common vetch seeds (8.78 log_10_ CFU mL^−1^) in a ratio of 20:1, fermented for 8 h at 37 °C, and stirred every 1 h. After that, the starch slurry was centrifuged at 5000× *g* for 25 min, and the supernatant was discarded. The precipitate was washed 4 times with distilled water and the wet starch was obtained through a 0.074 mm sieve. Finally, the common vetch starch was obtained by drying the wet starch in a hot air stream (DHG-9070A, Jintan, China) for 6 h at 45 °C.

Common vetch, pea starch, and mung bean starch gels were prepared according to the method of Rong Liyuan et al. [17]. Briefly, starch (10%, *w*/*v*) was dispersed into distilled water, and then the starch slurry was heated to 95 °C at a speed of 7.5 °C/min, maintained for 5 min, and then cooled to 50 °C using Rapid Visco-Analyzer (RVA, Newport Scientific, NSW, Australia). The gel sample was obtained for the testing of dynamic rheological properties, and some gels were transferred to plastic molds (20 mm × 20 mm × 20 mm) for 12 h at 4 °C for texture testing. In addition, some gels were frozen at −80 °C, then dried with a freeze dryer for in vitro digestibility analysis.

### 2.2. Physicochemical Compositions

Moisture, lipid, and protein content were determined according to the American Society of International Grain Chemists (AACCI) and Association of Official Analytical Chemists (AOAC) methods. The total starch content and amylose content were analyzed using an enzymatic method (total starch and amylose/amylopectin starch assay kit, Megazyme International Ireland Ltd., Bray, Ireland).

### 2.3. Starch Morphology and Particle Size Distribution

The morphological structure of starch granules was determined with scanning electron microscopy (GeminiSEM 500, ZEISS); samples were viewed and photographed using the SEM at an acceleration voltage of 10.0 kV and a magnification of 500× and 1000×.

The starch particle size distribution was detected with dynamic light scattering Mastersizer 2000 (Malvern Instruments Ltd., Malvern, UK). The samples were uniformly dispersed in distilled water, and the refractive index and absorption were set to 1.52 and 0.1, respectively [18].

### 2.4. Molecular Weight and Chain-Length Distributions

The homogeneity and molecular weight of starch were measured using SEC-MALLS-RI [19]. The weight- and number-average molecular weight (Mw and Mn) and polydispersity index (Mw/Mn) of starch in DMSO/LiBr (0.5% *w*/*w*) solution were measured on a DAWN HELEOS-II laser photometer (He-Ne laser, λ = 663.7 nm, Wyatt Technology Co., Santa Barbara, CA, USA) equipped with three tandem columns (300 × 8 mm, Shodex OH-Pak SB-805, 804, and 803; Showa Denko K.K., Tokyo, Japan), which were kept at 60 °C using a model column heater. The flow rate was 0.3 mL/min. A differential refractive index detector (Optilab T-rEX, Wyatt Technology Co., Santa Barbara, CA, USA) was simultaneously connected to give the concentration of fractions and the dn/dc value. Data were acquired and processed using ASTRA6.1 (Wyatt Technology).

The chain length distributions of starch were analyzed using high-performance anion-exchange chromatography with a CarboPac PA-100 anion-exchange column and a pulsed amperometric detector (HPAEC-PAD, Dionex ICS 5000 system, Thermo Fisher Ltd., Waltham, MA, USA) [20]. The pretreatment method of starch samples and the parameter settings of HPAEC-PAD adopted the method of Tang et al. [21].

### 2.5. X-ray Diffraction

The crystalline structure of starch was analyzed using a D8 Advance X-ray diffractometer (XRD) (Bruke AXS Ltd., Karlsruhe, Germany) with Ni-filtered Cu Ka radiation (35 kV, 20 mA). The X-ray diffractogram was acquired by scanning from the diffraction angle (2θ) of 4°–45° at a scanning speed of 3°/min and a step size of 0.02°. The relative crystallinity of starch was calculated as the same as Lopez-Rubio’s method [22]; the relative crystallinity of starch is the percentage of crystalline area relative to the total area.

### 2.6. Fourier Transform Infrared Spectroscopy (FT-IR) and Raman Spectroscopy

The FT-IR features of starch samples were measured using Fourier transform infrared spectrometer (Vertex 70, Bruker, Karlsruhe, Germany). Dry samples were prepared at a ratio of 1 g starch/100 g KBr. Signal collection was performed at a scan speed of 4 cm^−1^ over a scan range of 4000 cm^−1^–400 cm^−1^.

Raman spectra of starch samples were measured using a Renishaw Invia confocal Raman microscope system (Renishaw, Gloucestershire, UK) with a 785 nm green diode laser source [23]. Signal collection was performed at a scan speed of 7 cm^−1^ over a scan range of 3200 cm^−1^–100 cm^−1^.

### 2.7. Solid State ^13^C CP/MAS NMR Analysis

The ^13^C CP/MAS NMR analysis of starch was performed on a Bruker Avance III 400 WB spectrometer according to a previously established method [19]. And the calculation of the specific relative crystallinity (RC), double helix content (DH), and amorphous phase (PPA) adopted the method of Yin et al., and Atichokudomchai et al. [24,25].

### 2.8. Low-Field Nuclear Magnetic Resonance (LF-NMR) Measurement

The LF-NMR detection of the starch sample was performed on a Niumag benchtop pulsed NMR analyzer PQ001 (Nimag Electric Corporation, Shanghai, China) based on Shoaib’s method with slight modifications [26]. The resonance frequency, waiting time, time echo, and number of echoes were set to 21 MHz, 1000 ms, 0.4 ms, 10,000, and 8, respectively.

### 2.9. Pasting Property and Thermal Property

The pasting property of starch was investigated using a Rapid Visco Analyzer (RVA, Newport Scientific, NSW, Australia) [27]; a 10% (*w*/*w*, db) starch slurry was put into the RVA, and the starting temperature was set to 50 °C, then the temperature was raised to 95 °C at a speed of 7.5 °C/min, maintained for 5 min, and then cooled to 50 °C at the same speed, which was kept for 1 min.

The thermal property of starch was detected with differential scanning calorimeter (DSC) according to Wang et al., with slight modifications [28]. Briefly, the starch sample (2.5 mg, db) was mixed with deionized water (7.5 mL) and sealed in a DSC sample pan, and then equilibrated at room temperature for 24 h. The starting temperature was set to 30 °C, and the temperature was raised to 100 °C at a rate of 10 °C/min.

### 2.10. Starch Solubility and Swelling Power

The detection of starch solubility (SA) and swelling power (SP) was according to the method of Wang et al., with some modifications [29]. Starch solution (50 mL, 2%) was continuously stirred for 30 min in a water bath at 90 °C. After that, the samples were cooled to room temperature and centrifuged at 4000× *g* r/min for 20 min. The supernatant was transferred into an aluminum box and dried at 105 °C for 3 h. The solubility (SA) and swelling power (SP) were calculated according to the following equations:

SA (%) = m_1_/m × 100% (1)

SP (%) = m_2_/(m − m_1_) × 100% (2)

where m is the mass of starch that was put in at the beginning, m_1_ is the mass of starch in the aluminum box, and m_2_ is the mass of the precipitate after centrifugation.

### 2.11. Gel Dynamic Rheological Properties

Dynamic rheological properties of three starch gels were studied using an AR1500ex rheometer (TA Instruments, New Castle, DE, USA) with a plate geometry system (40 mm diameter and 1.000 mm gap). Frequency sweep tests were performed at frequencies of 0.1–10 Hz at 1% strain, the temperature was set as 25 °C, and the tests were performed in triplicate.

### 2.12. Gel Texture Properties

Texture properties of three starch gels were determined using the texture analyzer (TA-XT 2, Stable Micro System Co., Godalming, UK) equipped with a P30 probe. The pre-test speed, test speed, and post-test speed of the texture analyzer were set to 2.0 mm/s, 2.0 mm/s, and 2.0 mm/s, the strain was 50%, and the trigger force was 5 g. All tests were performed in triplicate.

### 2.13. In Vitro Digestibility Analysis of Gels

The 200 mg freeze-dried starch sample was taken for in vitro digestion simulation experiments. The specific experimental steps of the in vitro digestion referred to the method of Benavent-Gil et al. [30]; samples were taken at 0 min, 10 min, 20 min, 30 min, 45 min, 60 min, 90 min, 120 min, 180 min, and 240 min, respectively. The glucose content was determined using glucose oxidase–peroxidase (GOPOD, Megazyme International Ireland Ltd., Bray, Ireland) and absorbance was recorded at 510 nm. The amount of rapidly digestible starch (RDS), slowly digestible starch (SDS), and resistant starch (RS) fractions was determined using the method of Englyst et al. [31]; RDS was the fraction hydrolyzed within 20 min, SDS was the fraction hydrolyzed within 20 min to 120 min, and RS was the fraction unhydrolyzed after 120 min.

The hydrolysis index (HI) was obtained by dividing the area under the hydrolysis curve (0–180 min) by the area of the white bread sample over the same period, and the estimated glycemic index (GI) for the three starch gels was calculated using the method of García-Alonso et al. [32].

### 2.14. Statistical Analysis

Data statistics were calculated using SPSS v22.0 (SPSS, Chicago, IL, USA). The data are expressed as mean ± standard deviation and were statistically analyzed using Tukey’s multiple comparison test; the difference between the two groups is considered to be at the 95% level of significance (*p* < 0.05).

## 3. Results and Discussion

### 3.1. Physicochemical Compositions

As shown in Table 1, the contents of total starch, moisture, protein, and fat of the three starches were similar, but the amylose content of common vetch starch was significantly higher than that of mung bean starch and pea starch (*p* < 0.05); Fu et al., also found that some common vetch contained high amylose, such as CV5 (37.18 ± 0.13) and CV23 (38.68 ± 0.51) [8]. And the amylose content of common vetch starch was close to that of *Vicia faba* bean starch, which is a type of legume starch considered to be high in amylose [6]. These results indicate that common vetch starch had higher amylose content compared with pea and mung bean starch, and *Longjian* could be used as a pea resource with high amylose content.

### 3.2. Morphology Structure and Particle Distribution of Starch Granules

**Figure 1:**
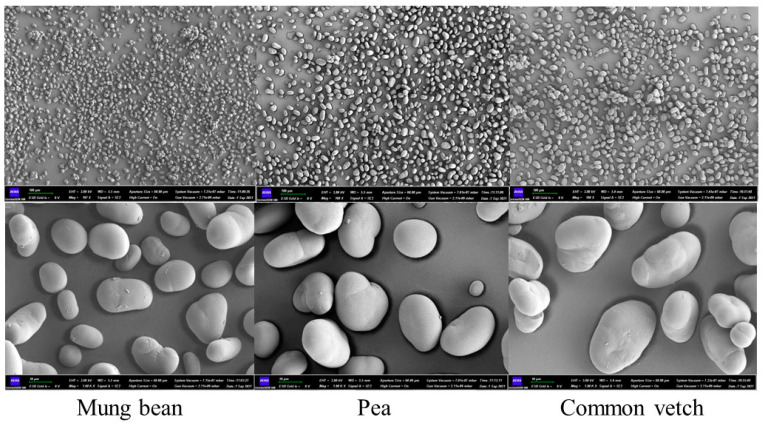
Scanning electron micrographs for common vetch, pea, and mung bean starches. Magnification of 100× and 1000×.

The appearance and size of the granules were close among the three starches. As shown in Figure 1, common vetch starch granules were spherical or ellipsoidal with a rough surface and relatively uniform size, mung bean starch granules were ellipsoidal, with a smooth surface and uniform size, and pea starch granules were ellipsoidal or irregularly spherical with folds on the surface and an uneven size. From the size of starch granules, common vetch starch had the largest particle size of 21.48–34.75 μm, followed by pea starch (16.12–28.90 μm) and mung bean starch (9.83–19.91 μm).

The particle size distributions of the three starches are presented in Table 2, and all of them showed a unimodal distribution. The particle size distribution of common vetch, the mung bean, and pea starch ranged from 18.31 to 43.56 μm, 9.54 to 27.10 μm, and 15.42 to 39.67 μm [33], respectively. The comparison of the values of D(4,3) and D(3,2) showed that the particle size of common vetch starch was significantly larger than that of pea starch and mung bean starch [34]. These results are in agreement with the sizes of starch granules observed with SEM.

### 3.3. Molecular Weight and Chain-Length Distributions

The molecular weight of starch was closely related to its thermal and digestive properties; the higher the molecular weight, the higher the crystallinity and the better the thermal stability [35]. The weight-average molecular weight (M_w_) and number-average molecular weight (M_n_) of the three starches are presented in Table 2; common vetch starch had the largest Mw and Mn, followed by mung bean starch and pea starch. The higher value of Mw/Mn and Rz of starch indicates larger differences in the molecular distribution or different sizes of particles [36]. The Mw/Mn values of common vetch starch, mung bean starch, and pea starch were 3.58, 3.50, and 2.35, respectively. The value of Rz represents the range of particles around the center and is consistent with the trend of the value of Mw/Mn. These results suggest that the common vetch starch had the highest degree of complexity and the highest degree of branching compared with mung bean and pea starches [35].

The chain length is divided into the following four parts: the A chain (DP 6–12), B1 chain (DP 13–24), B2 chain (DP 25–36), and B3 chain (DP ≥ 37) [37]. As shown in Table 2, the A chain and B1 chain of common vetch starch account for the largest proportion and the lowest proportion, respectively, compared to mung bean starch and pea starch. In addition, common vetch starch contained a higher proportion of B3 chains compared to mung bean starch. These results suggest that the short-chain structure of common vetch starch was more complex than that of the mung bean and pea starch, which may induce differences in the crystallinity and digestibility of starch gels.

### 3.4. Crystalline Structure

**Figure 2:**
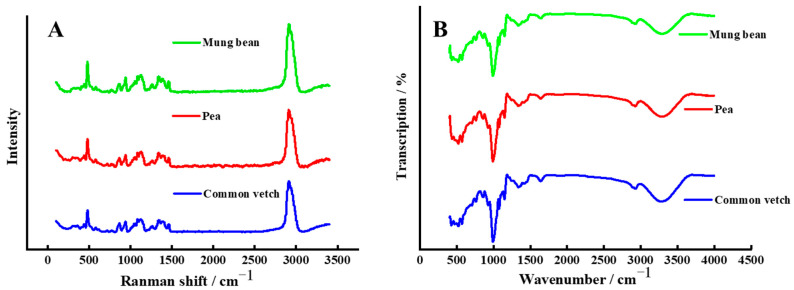
(**A**) is the result of the Raman spectrum for three starches and (**B**) is the result of FT-IR deconvoluted spectra for three starches.

**Figure 3:**
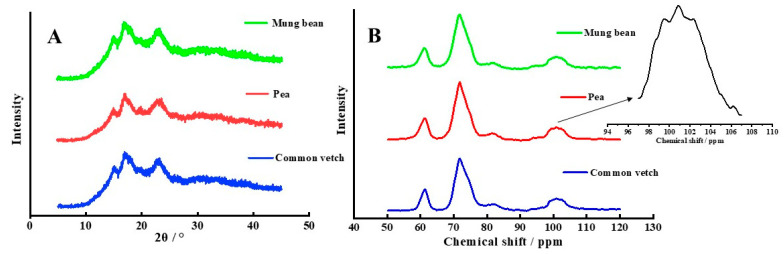
(**A**) is the result of XRD diffraction patterns for three starches and (**B**) is the result of ^13^C CP/MPS NMR spectra for three starches.

The crystal structure of starch is shown in Table 3 and Figure 2 and Figure 3. As shown in Figure 3A, the common vetch starch, mung bean starch, and pea starch present three obvious peaks at a 2θ value of 15°, 17°, and 23°, indicating a typical crystalline structure of the C-type. However, the crystallinity of common vetch starch was significantly higher than that of mung bean starch and pea starch (*p* < 0.05). This may be related to the size of starch granules and chain length distribution; larger starch granules predicted higher crystallinity [24,34,38], and the starch with a higher proportion of the A chain length had a higher relative crystallinity [38].

FT-IR is a well-established tool for monitoring the structure of macromolecular polymers. As can be seen from Figure 2B, the functional groups and chemical bonds of common vetch starch were similar to those of the mung bean and pea starch, since similar absorption bands were detected in the three starches. However, as shown in Table 3, the value of the degree of order (DO, 1047/1022 cm^−1^) and degree of double helix structure (DD, 995/1022 cm^−1^) of common vetch starch was close to that of mung bean starch and significantly higher than that of pea starch (*p* < 0.05), which indicate the short-range molecular order and long-range order of common vetch starch were similar to those of mung bean starch but higher than those of pea starch [15,34].

The short-range molecular order of the double helix of the starch sample can also be characterized by the FWHM value of the Raman band at 480 cm^−1^ [23]; smaller FWHM values indicate higher relative crystallinity in starch. As shown in Table 3, the FWHM value of the Raman band at 480 cm^−1^ in common vetch was significantly lower than that of mung bean starch and pea starch (*p* < 0.05), which is consistent with the results of relative crystallinity.

The ^13^C cross-polarization/magic angle spinning NMR is an advantageous way to resolve the helical and crystal structure of starch, especially the short-range ordered structure of starch molecules. As shown in Figure 3B and Table 3, the spectral signals of the three starches were similar and showed a weak triple C1 spectrum, suggesting that the starches were the C-type crystallite [39]. And the relative crystallinity detected with ^13^C-NMR of common vetch was the highest, followed by mung bean starch and pea starch, which is consistent with the result of XRD. However, the relative crystallinity obtained with ^13^C-NMR (36.35–47.32%) was greater than that obtained with XRD (27.31–34.07%), which may be because the crystallinity detected with ^13^C-NMR included crystalline layers and semi-crystalline regions [24,40]. In addition, the content of the double helix (DH) in common vetch starch was significantly higher than that of mung bean starch and pea starch (*p* < 0.05), which is consistent with the results of XRD and Raman measurements, indicating that the structural difference between the three starches was mainly related to the long-range crystalline and water-binding ability.

### 3.5. Hydrated Structure

**Figure 4:**
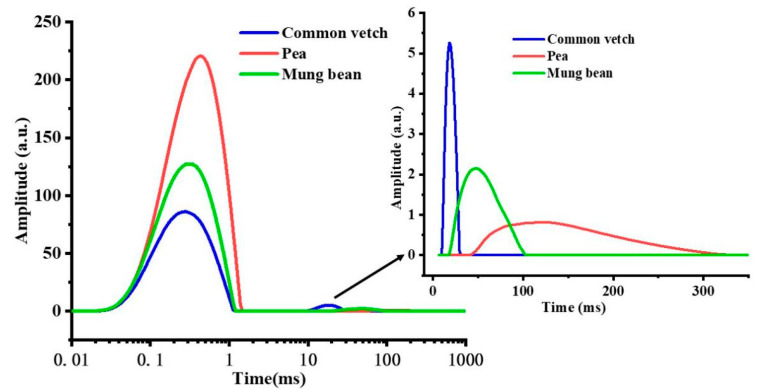
The free water/T_23_ (100–1000 ms), immobilized water/T_22_ (10–100 ms), and bound water/T_21_ (0.01–10 ms) of three starches obtained using LF-NMR.

Low-field nuclear magnetic resonance (LF-NMR) is used to determine the water-binding ability of macromolecular substances [41]. The quantification and distribution of water content were defined with rotational relaxation (T_2_) [26]. And a shorter T_2_ signal in a rigid or less mobile environment indicates that water was strongly bound and less active [42,43]. As shown in Figure 4 and Table 3, the relaxation peaks of the three starches were mainly at 0.01–1 ms (T_21_), indicating that the moisture of the three types of starches was mainly bound to water. However, the signal of T_21_ and the peak area A_21_ of common vetch starch were significantly lower than those of mung bean starch and pea starch (*p* < 0.05), indicating that the water binding ability of starch granules was weakest in pea starch and strongest in common vetch starch. These results also suggest that more stable hydrated structures were formed in common vetch starch compared to mung bean starch and pea starch.

### 3.6. Pasting and Thermal Properties

The pasting properties of common vetch, pea, and mung bean starches are shown in Table 4; the peak viscosity (PV), trough viscosity (TV), breakdown (BD), and setback (SB) values of common vetch starch were significantly lower (*p* < 0.05) than those of mung bean starch and pea starch, but its PT value was close to that of mung bean starch and significantly higher than that of pea starch. This may be related to the higher molecular weight, amylose content, and A-chain proportion of common vetch starch. Studies found that amylose content was significantly negatively correlated with the PV of starch, and low swelling extent also predicted a low viscosity value and BD value [44,45,46]. And an obvious negative correlation between the proportion of the A chain of starch and the PV value was concluded by Tong et al. [47]. In addition, Oyeyinka et al., found that larger starch granules resulted in smaller specific surface areas and lower pasting viscosity [44]. The higher PT value of common vetch starch may be due to its higher relative crystallinity and more double-helical content compared with pea starch. In addition, the lower BD and SB of common vetch starch compared with mung bean starch and pea starch indicate that common vetch starch had better hot-paste stability and cold-paste stability [46], which suggests that common vetch starch could be mixed with other starches as a stabilizer for starch processing.

Generally, T_o_ and Tc reflect the perfect degree of double helix structure or crystalline structure in starch granules, and the change of the enthalpy value of starch is the process of breaking the double helix structure and crystal structure under heating conditions [46,48]. As shown in Table 4, the To value of common vetch starch was significantly higher than that of mung bean starch and pea starch (*p* < 0.05), while the Tp value was close to that of mung bean starch and pea starch, and the Tc value was lower than that of mung bean starch and higher than that of pea starch, respectively. In addition, the ΔH of common vetch starch was significantly higher than that of mung bean starch and pea starch, and Fu et al., also found that the ΔH of CV35, CV12, and CV17 starches was significantly higher than that of smooth pea starches [8]. These results are consistent with the results of the crystallinity and double helix structure of the three starches.

### 3.7. Rheological Properties of Starch Gels

**Figure 5:**
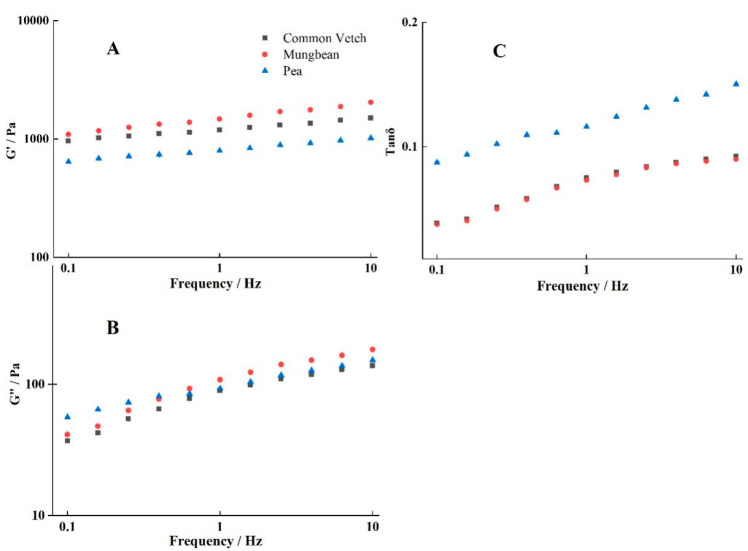
(**A**) is the result of storage modulus (G′), (**B**) is the result of loss modulus (G″), and (**C**) is the result of loss tangent (tan δ = G″/G′) for three starch pastes.

G′, G″, and tan δ as functions of frequency for different starch gels are presented in Figure 5; all the starch gels exhibited higher G′ than G″, behaving as viscoelastic solids, which is consistent with the results obtained with bitter vetch starch [4]. And both G′ and G″ increased with the increase in frequency, which may be due to the formation of small network structures with increasing deformation [49]. Linear regression of the dynamic rheological data of ln G′ and ln G″ versus ln Frequency is presented in Table S1; G″ (slope = 0.215–0.391) showed a much greater dependence on frequency than G′ (slope = 0.092–0.136). As shown in Figure 5C, tan δ of common vetch was lower than 0.1 and similar to that of the mung bean, but tan δ of the pea was higher than 0.1. And the G′ values of the systems were in descending order: mung bean > common vetch > pea. These results indicate that the common vetch starch gel was stronger than the pea starch gel and weaker than the mung bean starch gel [50].

### 3.8. Starch Solubility (SA), Swelling Power (SP)

**Figure 6:**
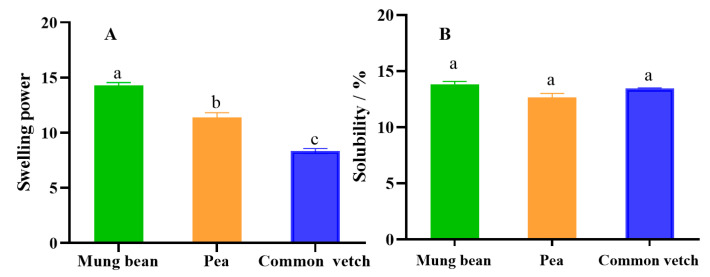
(**A**) is the result of solubility for three starches, and (**B**) is the result of swelling power for three starches, where values with different letters are significantly different (*p* < 0.05).

The solubility and swelling power of the three starches are presented in Figure 6A,B. The solubility of common vetch starch was higher than that of mung bean starch and pea starch, but the swelling power of common vetch starch was significantly lower than that of mung bean starch and pea starch (*p* < 0.05). This may be related to the content of amylose, molecular weight, branching degree, and chain length distribution [51]. Some studies illustrated that the content of amylose had a positive correlation with the SA of starch and a negative correlation with the SP [52], and the larger the molecular weight and granules, the higher the solubility and the lower the swelling degree [53]. These results also showed that the internal structure of the common vetch was more compact than that of the mung bean and pea starch.

### 3.9. Texture Properties and In Vitro Digestion of Starch Gels

Texture properties are a necessary feature of starch-based foods. The hardness of starch gel is mainly caused by the network structure formed by the retrogradation of starch, which is related to the content of amylose, the viscosity of starch, and the crystallinity of amylopectin [54]. Springiness reflects the recovery of starch gels, and cohesiveness represents the difficulty of breaking starch gel, which is related to the degree of structure of starch gel [55]. Gumminess is related to the firmness of starch and reflects the energy required to break down starch gels into semi-solid foods [56]. As shown in Table 5, the hardness, springiness, and gumminess of common vetch starch gel were lower than mung bean starch gel, which may be due to the smaller particles and higher viscosity of mung bean starch [57]. And the TPA properties of common vetch starch gel were higher compared to pea starch gel, which may be caused by the higher amylose content and crystallinity of vetch starch. Studies have shown that starch with high amylose content is prone to short-term retrogradation and forms compact structures [56]. These results indicated common vetch starch was a better ingredient for chewy foods than pea starch.

**Figure 7:**
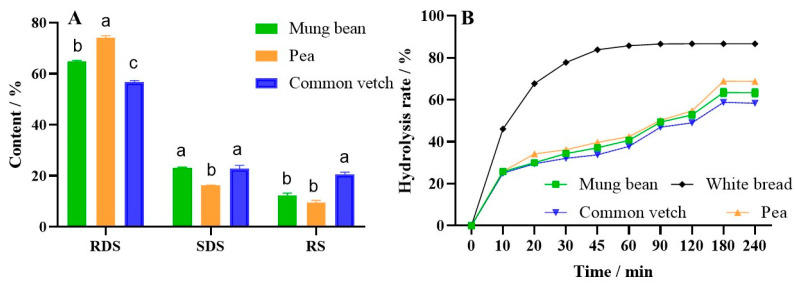
(**A**) is the result of rapidly digestible starch (RDS), slowly digestible starch (SDS), and resistant starch (RS) content, and (**B**) is the result of hydrolysis of three starches, where values with different letters are significantly different (*p* < 0.05).

The content of rapidly digestible starch (RDS), slowly digestible starch (SDS), and resistant starch (RS) in three starch gels is shown in Figure 7A and Table 5; the RDS and RS content of vetch starch was significantly lower and higher than that of mung bean starch and pea starch, respectively, while the SDS content was close to that of mung bean starch and significantly higher than that of pea starch. These results illustrate that a higher structure was conformed in common vetch starch gel, and studies found that the high amylose content and the high proportion of A chains could induce the formation of intermolecular hydrogen bonds, double helixes, and supramolecular structures during starch gelatinization [58].

As shown in Figure 7B and Table 5, the HI and GI values of common vetch starch gel were lower than those of the mung bean and pea starch gel, which was due to its higher content of resistant starch and slowly digestible starch [15], which is also consistent with the results of cohesiveness. Studies have also shown that starch molecules with a higher proportion of A chains tend to have higher retrogradation rates and faster intermolecular and intramolecular interactions, resulting in lower starch gel digestibility [59,60,61]. These results also suggested that common vetch starch had better slow-digesting properties compared with mung bean starch and pea starch, and it could be used as a low-glycemic starch material.

## 4. Conclusions

The composition and chemical structure of common vetch starch were not significantly different from the commercial pea and mung bean starch. But the amylose content of common vetch starch was considerably higher than that of the commercial pea and mung bean starch (*p* < 0.05). And the granules and particle size distributions of common vetch starch were larger than those of mung bean starch and pea starch. The short-range order degree of common vetch starch was similar to that of mung bean starch but higher than that of pea starch, while the relative crystallinity of common vetch starch was significantly higher than that of mung bean starch and pea starch. Compared with mung bean starch and pea starch, the gelatinization of common vetch starch required more energy, and the cold-paste stability and hot-paste stability were higher, but the pasting viscosity was lower. In addition, common vetch starch had better solubility and lower swelling power, and the gel viscosity and texture properties of common vetch starch were higher than those of pea starch. More resistance to digestion was found in common vetch starch gel than in mung bean starch and pea starch gels. The results indicate that common vetch starch can be used as an excellent compound for starch processing to improve the stability and slow digestibility of starch products. More research related to the product processing of common vetch starch is needed to expand its application fields.

## Figures and Tables

**Table 1:** Physicochemical compositions of common vetch, pea, and mung bean starches.

	Mung Bean Starch	Pea Starch	Common Vetch Starch
Total starch	91.22 ± 0.27 ^a^	91.79 ± 0.19 ^a^	92.56 ± 0.39 ^a^
Moisture	4.71 ± 0.03 ^b^	5.67 ± 0.03 ^a^	4.85 ± 0.05 ^b^
Lipid	0.93 ± 0.021 ^a^	0.93 ± 0.01 ^a^	0.77 ± 0.12 ^b^
Protein	0.93 ± 0.12 ^a^	1.03 ± 0.09 ^a^	0.83 ± 0.12 ^a^
Amylose	29.98 ± 0.22 ^b^	27.46 ± 0.54 ^c^	35.69 ± 0.54 ^a^

Values with different letters within a row are significantly different (*p* < 0.05).

**Table 2:** Particle size distribution, molecular weight, and chain length distribution of common vetch, pea, and mung bean starches.

	Mung Bean Starch	Pea Starch	Common Vetch Starch
D(4,3)	17.49 ± 0.03 ^c^	26.35 ± 0.01 ^b^	29.28 ± 0.03 ^a^
D(3,2)	9.16 ± 0.01 ^c^	13.58 ± 0.01 ^b^	26.44 ± 0.02 ^a^
D10	9.54 ± 0.01 ^c^	15.42 ± 0.01 ^b^	18.25 ± 0.02 ^a^
D50	17.07 ± 0.02 ^c^	25.91 ± 0.01 ^b^	27.85 ± 0.02 ^a^
D90	27.10 ± 0.05 ^c^	39.67 ± 0.03 ^b^	42.42 ± 0.04 ^a^
Mw (kDa)	39,310 ± 89 ^b^	24,996 ± 57 ^c^	44,042 ± 149 ^a^
Mn(kDa)	11,250 ± 244 ^a^	10,625 ± 192 ^b^	12,309 ± 128 ^c^
Mw/Mn	3.50 ± 0.08 ^a^	2.35 ± 0.05 ^b^	3.58 ± 0.03 ^a^
Rz (nm)	138 ± 1.4 ^b^	116 ± 0.5 ^c^	149 ± 0.3 ^a^
DP 6–12 (%)	35.05 ± 0.11 ^b^	33.76 ± 0.37 ^c^	37.62 ± 0.04 ^a^
DP 13–24 (%)	45.58 ± 1.38 ^a^	44.86 ± 1.23 ^a^	40.84 ± 0.05 ^b^
DP 25–36 (%)	11.62 ± 0.36 ^a^	12.25 ± 0.20 ^a^	11.96 ± 0.01 ^a^
DP > 37 (%)	7.76 ± 0.94 ^b^	9.11 ± 0.65 ^a^	9.58 ± 0.01 ^a^

Values with different letters within a row are significantly different (*p* < 0.05).

**Table 3:** Short-range molecular order and long-range crystalline structure of common vetch, pea, and mung bean starches with X-ray diffraction, FR-IR, Raman spectrum, and ^13^C-NMR; moisture distribution (T_2_) and content (A, peak area) detected with LF-NMR.

	Mung Bean Starch	Pea Starch	Common Vetch Starch
XRD			
Crystal type	C	C	C
RC1 (%)	31.49 ± 0.70 ^b^	27.61 ± 0.29 ^c^	34.16 ± 0.20 ^a^
FT-IR			
DO (1047 cm^−1^/1022 cm^−1^)	1.39 ± 0.00 ^a^	1.33 ± 0.00 ^b^	1.41 ± 0.01 ^a^
DD (995 cm^−1^/1022 cm^−1^)	0.88 ± 0.00 ^a^	0.77 ± 0.00 ^b^	0.87 ± 0.01 ^a^
Raman spectrum			
FWHM (480 cm^−1^)	29.68 ± 0.70 ^b^	32.29 ± 0.41 ^a^	24.19 ± 0.60 ^c^
^13^C-NMR			
C1 (ppm)	103.25, 100.65, 99.01	103.93, 100.80, 99.19	103.43, 101.15, 99.22
C2, 3, 5 (ppm)	76.19, 74.06, 71.55	75.52, 73.67, 71.55	76.13, 74.39, 71.60
C4 (ppm)	81.56	81.23	81.27
RC2 (%)	43.8 ± 1.56 ^b^	36.35 ± 1.23 ^c^	51.29 ± 1.11 ^a^
DH (%)	59.14 ± 0.86 ^b^	55.64 ± 0.77 ^c^	62.88 ± 1.03 ^a^
PPA (%)	6.54 ± 0.31 ^b^	10.13 ± 1.02 ^a^	4.72 ± 0.22 ^c^
LF-NMR			
A21	3712 ± 76 ^b^	6300 ± 280 ^a^	2689 ± 71 ^c^
T21 (%)	99.12 ± 0.6 ^a^	99.76 ± 0.4 ^a^	98.21 ± 0.04 ^b^
A22	32.73 ± 2.12 ^b^	6.73 ± 0.32 ^c^	48.96 ± 0.01 ^a^
T22 (%)	0.87 ± 0.04 ^b^	0.11 ± 0.06 ^c^	1.79 ± 0.04 ^a^
A23	0	8.19 ± 0.18	0
T23 (%)	0	0.13 ± 0.02	0

Values with different letters within a row are significantly different (*p* < 0.05).

**Table 4:** Pasting properties, thermal properties, swelling power, solubility, and in vitro digestion indicators of common vetch, pea, and mung bean starches.

	Mung Bean Starch	Pea Starch	Common Vetch Starch
PV (cP)	4461 ± 45 ^a^	2770 ± 31 ^b^	1905 ± 17 ^c^
TV (cP)	2504 ± 25 ^a^	2100 ± 23 ^b^	1553 ± 14 ^c^
BD (cP)	1957 ± 20 ^a^	670 ± 8 ^b^	352 ± 3 ^c^
FV (cP)	4289 ± 43 ^a^	3969 ± 44 ^b^	2762 ± 25 ^c^
SB (cP)	1784 ± 18 ^b^	1869 ± 21 ^a^	1208 ± 11 ^c^
PT (°C)	75.92 ± 0.25 ^a^	74.3 ± 0.07 ^b^	75.6 ± 0.12 ^a^
To (°C)	55.72 ± 0.28 ^a^	56.75 ± 0.13 ^b^	57.46 ± 0.14 ^c^
Tp (°C)	68.79 ± 0.09 ^a^	68.66 ± 0.54 ^a^	67.59 ± 0.99 ^a^
Tc (°C)	83.02 ± 0.64 ^a^	77.04 ± 0.71 ^c^	80.50 ± 0.19 ^b^
ΔH (J/g)	17.43 ± 0.23 ^b^	15.00 ± 0.99 ^c^	20.17 ± 0.31 ^a^
SA (%)	13.84 ± 0.18 ^a^	12.66 ± 0.26 ^b^	14.03 ± 0.23 ^a^
SP	14.56 ± 0.01 ^a^	11.19 ± 0.12 ^b^	8.11 ± 0.07 ^c^

cP: centipoise; PV: peak viscosity; TV: trough viscosity; BD: breakdown (BD = PV − TV); FV: final viscosity; SB: setback (SB = FV − TV); PT: pasting temperature. To: onset temperature; Tp: peak temperature; Tc: conclusion temperature; ΔH: enthalpy change of gelatinization. Values with different letters within a row are significantly different (*p* < 0.05).

**Table 5:** Texture properties and in vitro digestion indicators of common vetch, pea, and mung bean starch gels.

	Mung Bean Starch	Pea Starch	Common Vetch Starch
Hardness (N)	11960 ± 95 ^a^	4201 ± 46 ^c^	4536 ± 86 ^b^
Springiness	6.35 ± 0.05 ^a^	3.78 ± 0.02 ^c^	5.88 ± 0.10 ^b^
Cohesiveness	0.29 ± 0.00 ^b^	0.23 ± 0.00 ^c^	0.42 ± 0.00 ^a^
Gumminess	38.12 ± 0.52 ^a^	7.18 ± 0.06 ^c^	8.53 ± 0.13 ^b^
RDS (%)	64.39 ± 0.30 ^b^	74.21 ± 0.04 ^a^	56.78 ± 0.1 ^c^
SDS (%)	22.85 ± 0.14 ^a^	16.22 ± 0.05 ^b^	22.15 ± 0.58 ^a^
RS (%)	12.75 ± 0.04 ^b^	9.57 ± 0.01 ^c^	21.07 ± 0.48 ^a^
HI	49.10 ± 0.73 ^b^	52.61 ± 1.22 ^a^	43.28 ± 1.13 ^c^
GI	66.66 ± 0.47 ^b^	68.59 ± 0.31 ^a^	63.47 ± 0.24 ^c^

Values with different letters within a row are significantly different (*p* < 0.05).

## Data Availability

The data presented in this study are available in this article.

## Supplementary Materials

The following supporting information can be downloaded at: https://www.mdpi.com/article/10.3390/foods12132513/s1, Table S1: Slopes (Pa·s) and intercepts (Pa) of ln (G′, G″) versus ln Frequency (Hz) data of three starch paste at 25 °C.
