# Supplementary material for: Multi-Scale Comparison of Physicochemical Properties, Refined Structures, and Gel Characteristics of a Novel Native Wild Pea Starch with Commercial Pea and Mung Bean Starch
Source: Foods. 2023 Jun 28;12(13):2513. doi: 10.3390/foods12132513 (PMC10341203; doi:10.3390/foods12132513)
Supplement: Supplementary file 1 [file foods-12-02513-s001.zip › foods-2444479-supplementary.pdf]

Table S1. Slopes (Pa·s) and intercepts (Pa) of ln (G‘, G“) versus ln Frequency (Hz) data of three starch paste at 25 °C.

Samples	G‘			G“		
	Slope	Intercept	R^2	Slope	Intercept	R^2
Common vetch	0.092 ± 0.001	3.078 ± 0.006	0.99	0.391 ± 0.019	1.943 ± 0.014	0.98
Mung bean	0.136 ± 0.003	3.170 ± 0.004	0.99	0.295 ± 0.020	2.022 ± 0.006	0.96
Pea	0.099 ± 0.001	2.909 ± 0.008	0.99	0.215 ± 0.003	1.971 ± 0.002	0.99

Mean values ± standard deviation of triplicates
